# Supplementary material for: Hypoglycemic Effects and Quality Marker Screening of Dendrobium nobile Lindl. at Different Growth Years
Source: Molecules. 2024 Feb 2;29(3):699. doi: 10.3390/molecules29030699 (PMC10856227; doi:10.3390/molecules29030699)
Supplement: Supplementary file 1 [file molecules-29-00699-s001.zip › molecules-2820845-supplementary.pdf]

Supplementary materials

1.Methodological validation

1.1 Herb Pre-treatment

The freshly harvested stems and leaves of *D. nobile* were separated, washed, air-dried, weighed for fresh weight, cut into sections of 1-2 cm, dried at 60°C, pulverized after being sieved through a 50-mesh sieve.

1.2 Determination of Dendrobine Content by LC-MS

1.2.1 Preparation of standard reference solution

Accurately weigh 5 mg of dendrobine reference substance, make up with methanol, to prepare a stock solution of dendrobine reference substance with a concentration of 5 μg/mL.

Accurately weigh 5 mg of pseudoephedrine reference substance, make up with methanol, to prepare a stock solution of pseudoephedrine internal standard with a concentration of 1 μg/mL.

1.2.2 Preparation of Sample Solution

Precisely weighed samples of the powdered form of *D. nobile* with different growth years (S1,S7), each weighing 2 g, were added to 10 mL of 95% ethanol solution. The mixture was extracted by heating and refluxing three times, each time for 2 hours. The filtrate was consolidated and concentrated at reduced pressure to obtain the residue, which was then subjected to vacuum freeze-drying. A precisely weighed amount of 10 mg of the freeze-dried powder was diluted with methanol to a final volume of 10 mL and then centrifuged at 12 000 rpm for 5 minutes. A precisely measured amount of 0.5 mL of the supernatant was used for testing, with pseudoephedrine as the internal standard. A precise amount of 3.2 mL of pseudoephedrine stock solution (final concentration of 320 ng/mL) was added to the sample and mixed absolutely with methanol. After passing through a 0.22 μm organic microporous membrane filter, the sample was ready for testing.

1.2.3 Analysis Conditions for Determination of Dendrobine Content

The determination of dendrobine content was performed on the SCIEX LC-MS system, using a Poroshell 120 EC-C18 column (3.0 100 mm, 2.7 μm) as the stationary phase and a gradient elution with 0.1% formic acid water (A) - acetonitrile (B) as the mobile phase. The elution conditions are described in detail in Table S1. The flow rate was set at 0.3 mL/min, the column temperature was maintained at 30℃, and the injection volume was 2 μL. The mass spectrometry employed the positive ion multiple reaction monitoring (MRM) mode, with electrospray ionization mass spectrometry (ESI) ionization. The ion spray voltage (IS) was set at 5500 V, the curtain gas (CUR) was set at 25 psi, the ion source gas 1 (GS1) was set at 40 psi, the ion source gas 2 (GS2) was set at 45 psi, and the temperature (TEM) was set at 450℃. The collision gas (CAD) was set at 8 psi. The collision energy for dendrobine was 55 V, and for pseudophedrine it was 15 V.

Table S1 The gradient elution conditions for the determination of dendrobine content

Time (min)	0.1% formic acid in water (A%)	Acetonitrile (B%)
0	90	10
1	90	10
3	75	25
4	75	25
5	90	10
9	90	10

1.2.4 Detection Limit and Quantification Limit

The reference solution of dendrobine was diluted to different concentrations, namely 1, 3, 5, 10, and 15 ng/mL, filtered through a 0.22 μm organic microporous membrane, and analyzed under the conditions described in section *1.2.3*. The limit of detection (LOD) and the limit of quantification (LOQ) of dendrobine in this method

were determined using software Analyst MD 1.6.3 by calculating the signal-to-noise ratio (S/N) with S/N=3 and S/N=10 as the criteria.

1.2.5 Standard Curve and Its Linear Range

Precise amounts of dendrobine reference stock solution were taken, in accordance with the quantitation limit, the dendrobine reference stock solution was gradually diluted with methanol to prepare a series of solutions with dendrobine concentrations of 10, 20, 40, 80, 160, 320, 640, 1280, and 2560 ng/mL. The final concentration of pseudoephedrine internal standard was 320 ng/mL. Following the method outlined in section *1.2.3*, the dendrobine concentration was plotted on the x-axis, and the peak area ratio (Ar/As) of the reference peak area to the internal standard peak area was plotted on the y-axis to construct a standard curve.

1.2.6 Stability and Precision

Test solutions prepared according to the method under section *1.2.1* were analyzed at 0, 2, 4, 8, 12, and 24 hours under the conditions described in section *1.2.3*. The stability was evaluated by calculating the ratio of the peak area of Dendrobine at each time point to the internal standard peak area (Ar/As). The RSD value was then calculated. Control solutions of dendrobine at low, medium, and high concentrations within the linear range were continuously injected for 6 days under the analysis conditions described in section *1.2.3*. Three injections were made per day. The intra-day precision and inter-day precision were evaluated by calculating the ratio of the peak area of dendrobine to the internal standard peak area (Ar/As) for each injection. The RSD value was then calculated.

1.2.7 Reproducibility

Six samples of the same batch of *D. nobile* (S1) were accurately weighed. The test sample solution was prepared according to the method under *1.2.1*, and the content of dendrobine in the test sample was determined under the conditions of *1.2.3*, and the RSD value was calculated.

1.2.8 Sample recovery

Nine samples from the same batch of *D. nobile* (S1) were accurately weighed at 0.5g. In line with the content of dendrobine in each sample, add 80%, 100%, and 120% of the dendrobine reference substance respectively. Prepare the test solution according to the method in *1.2.1* and analyze it using the method in *1.2.3*. Calculate the sample recovery rate and RSD value using the following formula:

Sample recovery rate = (detected quantity - sample quantity) / added quantity × 100%

1.3 Determination of the Content of Sesquiterpene Glycosides by LC-MS

1.3.1 *Preparation of Reference Solution*

Accurately weighed 5 mg of DnA, DnC, DnD, DG, DmD, and DnE reference substance respectively, diluted with methanol to a final volume, and prepared a mixed reference solution of each reference substance at a concentration of 0.5 μg/mL.

1.3.2 Preparation of Sample Solution

Precisely weighed amount of 10 mg of the freeze-dried powder and diluted with methanol to a final volume of 10 mL and then centrifuged at 12,000 rpm for 5 minutes. A precisely measured amount of 0.5 mL of the supernatant was used for testing, Longdanxieganin was used as the internal standard (final concentration of 100 ng/mL). The mixture was made up to 10 mL with methanol, mixed well, filtered through a 0.22 μm organic microporous membrane, and then subjected to measurement.

1.3.3 Analysis conditions for determination of sesquiterpene glycosides content

The analysis conditions for determination of saponin content were established by other members of the research team and data are pending publication.

The content determination was performed on a SCIEX LC-MS using a Poroshell 120 EC-C18 (3.0 x 100 mm, 2.7 μm) chromatographic column as the stationary phase and water-acetonitrile as the mobile phase with a gradient elution at a flow rate of 0.3

mL/min and a column temperature of 30℃. The injection volume was set to 2 μL. The mass spectrometry was conducted in negative ion MRM mode with ESI ionization using nitrogen as nebulizer gas and the ionization voltage set at -5.5 kV, and the ion transfer tube temperature set at 450℃.

1.4 Screening of secondary metabolites with differential expression in D. nobile

1.4.1 Preparation of test solution

Precisely weighed 1g of *D. nobile* samples from different batches (detailed information is shown in Table 1, and added 10-fold volume of 95% ethanol-aqueous solution. The mixture was heated under reflux at 85° C for 2 h each time, repeated 3 times. The filtrates were combined and concentrated under reduced pressure to the same volume, to ensure the same content of crude drugs.

1.4.2 UPLC-QE/Orbitrap/MS Analysis Conditions

The UPLC-QE/Orbitrap/MS was used to analyze the secondary metabolites of *D. nobile*. The analysis was performed on a Hypersil GOLD C18 column (2.1 × 150 mm, 1.9 μm) as the stationary phase, with a mobile phase consisting of 0.1% formic acid solution (A) and acetonitrile (B). The gradient elution conditions are shown in Table S2, and the detection time ranged from 3.5 to 68 min. The flow rate was set at 0.3 mL/min, and the column temperature was maintained at 40℃. A sample volume of 3 μL was injected for analysis. The mass spectrometry technique used electrospray ionization (ESI) in positive ion mode. It employed a two-step scanning mode, with full scan at first and automatic triggering of secondary mass spectrometry scan. The scan range was set from 50 to 1500 Da. The electrospray voltage was maintained at 3.5 kV, and the ionization temperature was set at 300 ℃. The sheath gas flow rate was set at 35 arb, and the auxiliary gas flow rate was set at 15 arb. The capillary temperature was maintained at 350° C, and the collision energy was set at 25 V.

Table S2 The gradient elution conditions

Time(min)	0.1% formic acid in water（A%）	Acetonitrile（B%）
0	95	5
5	90	10
10	80	20
20	70	30
42	52	48
50	40	60
62	20	80
68	10	90
73	10	90
75	95	5
77	95	5

1.4.3 Stability and Precision

Take an appropriate amount of *D. nobile* samples (S1) and prepare the test solution following the method under section *1.4.1.* Place the solution at room temperature for 0, 2, 4, 6, 8, 16, and 24 hours. Then, under the conditions specified in section *1.4.2*, perform injections and determine the relative retention time and RSD value of the relative peak area for the main characteristic peaks, using the peak of dendrobine as a reference peak.

Take an appropriate amount of *D. nobile* powder samples (S1) and prepare the test solution following the method under section *1.4.1.* Perform consecutive injections 6 times under the conditions specified in section *1.4.2.* Using the peak of dendrobine that a common composition in the samples as a reference peak, evaluate the consistency of the relative retention time and relative peak area of the chromatographic peaks. Calculate the RSD value of the relative retention time and relative peak area for the main characteristic peaks.

1.4.4 Reproducibility

Take an appropriate amount of *D. nobile* powder sample (S1), prepare six parallel test sample solutions according to the method under *1.4.1,* and determine

them under the conditions specified in *1.4.2* by injection. Using the peak of dendrobine as a reference peak, calculate the RSD values of the relative retention time and relative peak area of the main characteristic peaks.

2. Methodological results

2.1 Chromatogram of the determination the content of dendrobine and sesquiterpene glycosides

As shown in Figure S1. The results showed that there was no interference at the peak of dendrobine and six sesquiterpene glycosides, and the resolution was good.

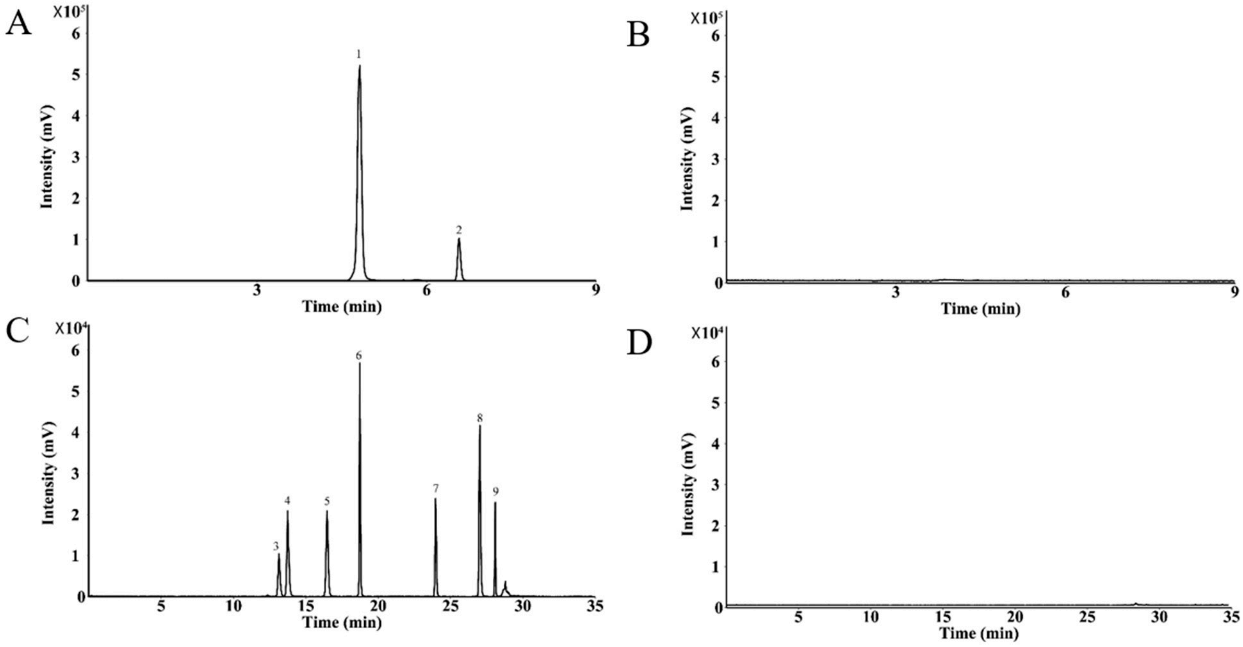

Figure S1. The chromatogram of dendrobine and six sesquiterpene glycosides

Notes: (A) Chromatogram of dendrobine control in positive ionization mode; (B) methanol chromatogram of blank solvent in positive ionization mode (C) chromatogram of sesquiterpene glycosides in negative ionization mode; (D) methanol chromatogram of blank solvent in negative ionization mode. Peak 1 was pseudoephedrine (internal standard), peak 2 was dendrobine, peak 3 was Dendromoniliside D, peak 4 was gentian bitteroside (internal standard), peak 5 was Dendronobiloside E, peak 6 was Dendroside G, peak 7 was Dendronobiloside D, peak 8 was Dendronobiloside C, Peak 9 was Dendronobiloside A.

2.2 Methodological review of dendrobine content determination

2.2.1 Limit of detection and quantification

According to the analysis methods as mentioned in *1.2.4*, using software Analyst MD 1.6.3 and a signal-to-noise ratio of S/N=3, the detection limit was found to be 3 ng/mL. The quantitation limit, calculated using S/N=10, was determined to be 10 ng/mL.

2.2.2 Standard curve and linear range

According to the analysis methods as mentioned in *1.2.5*, the standard curve was plotted by taking the dendrobine concentration as the X-axis and its corresponding peak-to-area ratio as the Y-axis. The results are shown in Figure S2, the equation is: $Y=3.37*10^{-4}X-1.53*10^{-4}$, $r^2=0.9991$, indicating good linearity in the concentration range of 10~2560 ng/mL.

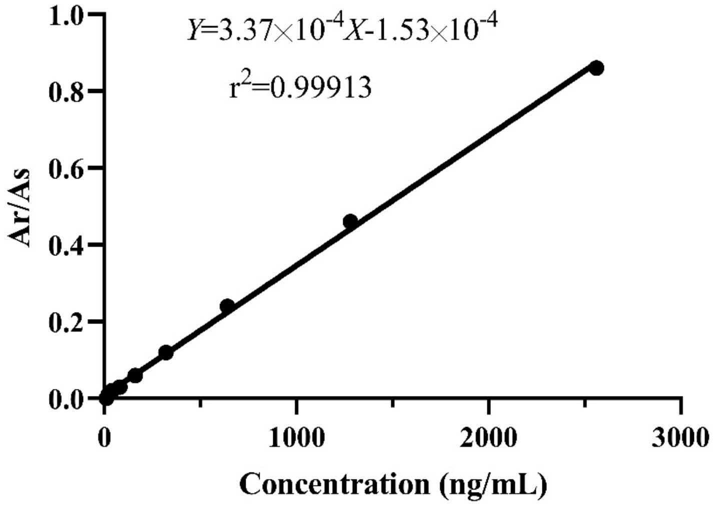

Figure S2. Standard curve for the determination of dendrobine content

2.1.3 Stability, precision and repeatability

The sample solution prepared according to the method as mentioned in *1.2.6* was determined by injection at 0, 2, 4, 6, 8, 12, 16 and 24 h according to the conditions in *1.2.3*, and the RSD was 2.22%, indicating that the method was stable well.

According to the conditions in *1.2.3*, the intraday and interday precision were reviewed, and the RSD < 2.74%, indicating that the precision was good.

The repeatability was examined according to the conditions in *1.2.3*, and the RSD was 2.40%, indicating that the method was reproducible.

2.1.4 Average recovery

According to the conditions in *1.2.3*, the recovery rates of low concentration, medium concentration and high concentration were investigated, and the values were 97.17%-102.46%, and the RSD < 2.30%, indicating that the accuracy of the method was good.

2.2 Methodological review of secondary metabolites with differential expression in D. nobile

2.2.1 Stability and Precision

According to the method described in section *1.4.3*, sample solutions were prepared and left at room temperature for 0, 2, 4, 6, 8, 16, and 24 hours. The solutions were then analyzed using the conditions described in section *1.4.2*, dendrobine used as a reference compound. The relative standard deviation (RSD) for relative retention time was 0.11%, and the RSD for relative peak area was 3.12%, indicating good stability of the sample solution within 24 hours.

Following the method described in section *1.4.3*, sample solutions were prepared and analyzed 6 times under the conditions described in section *1.4.2*. dendrobine was used as a reference compound and the RSD for relative retention time was 0.14%, while the RSD for relative peak area was 2.57%, indicating good precision of the instrument.

2.2.2 Reproducibility

Using the method described in section *1.4.4*, 6 sample solutions in parallel were prepared and analyzed under the conditions described in section *1.4.2*, dendrobine was used as a reference compound and the RSD for relative retention time was 0.12%,

while the RSD for relative peak area was 2.80%, indicating good reproducibility of the method.

3.The secondary mass spectra and fragmentation patterns of D. nobile

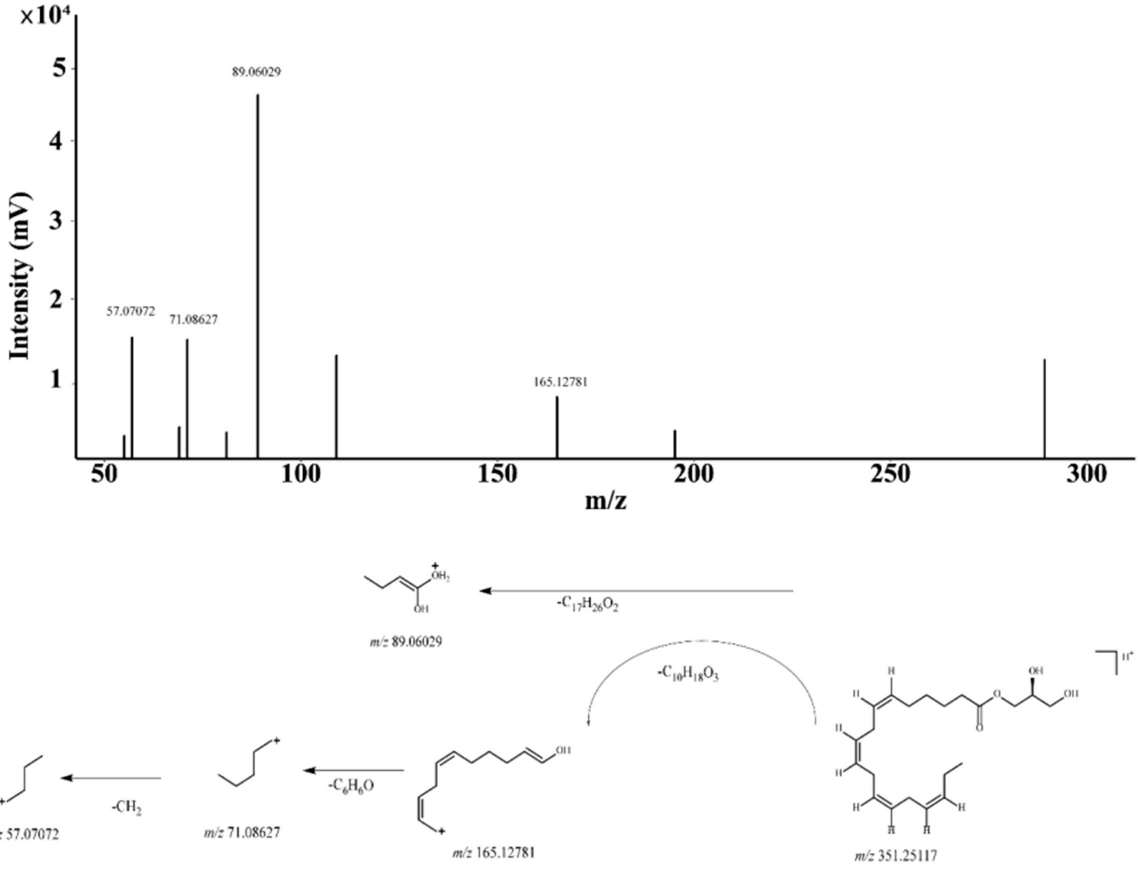

Figure.S3 The targeted MS/MS and fragmentation pattern of 1-Stearidonoyl-Glycerol

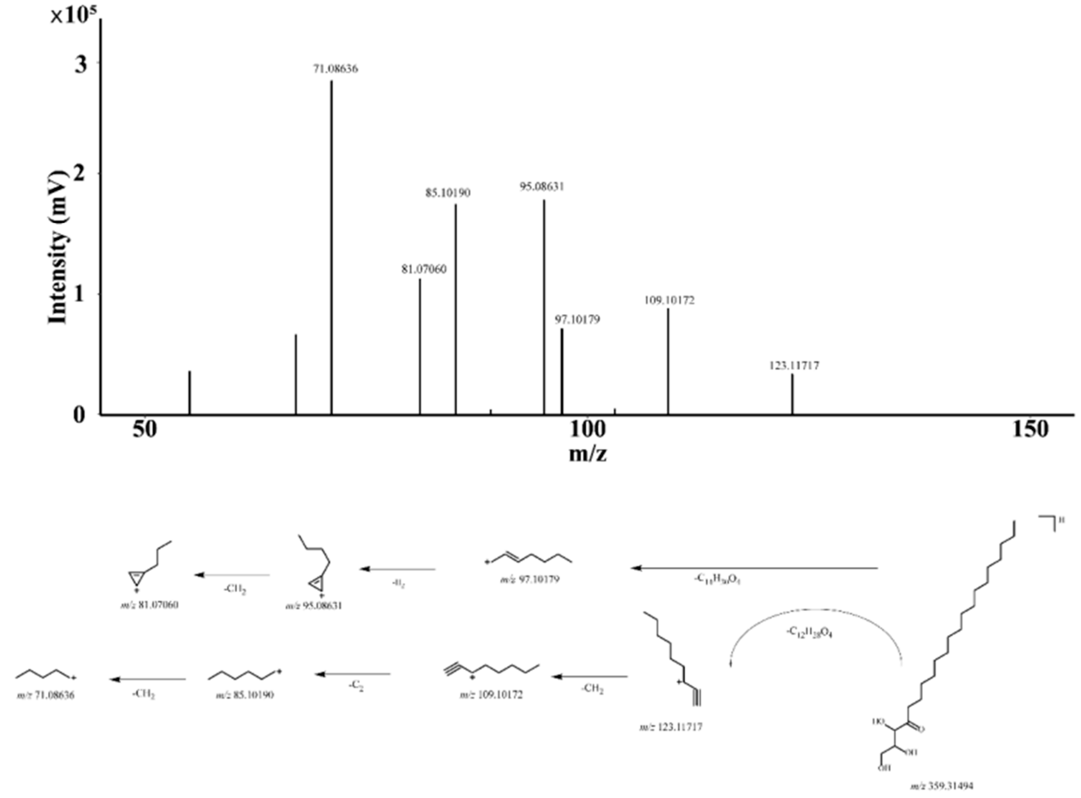

Figure.S4 The targeted MS/MS and fragmentation pattern of 1-Stearoylglycerol

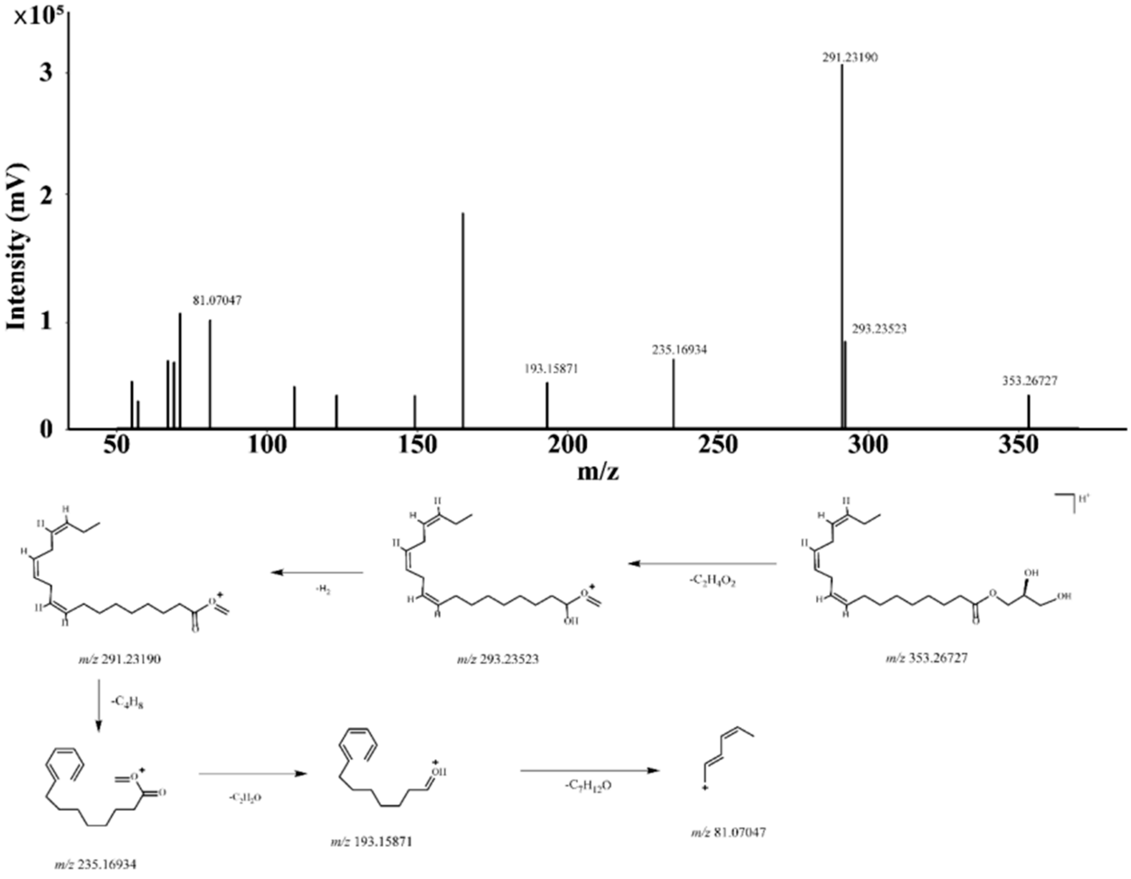

Figure.S5 The targeted MS/MS and fragmentation pattern of 1-α-Linolenoyl-glycerol

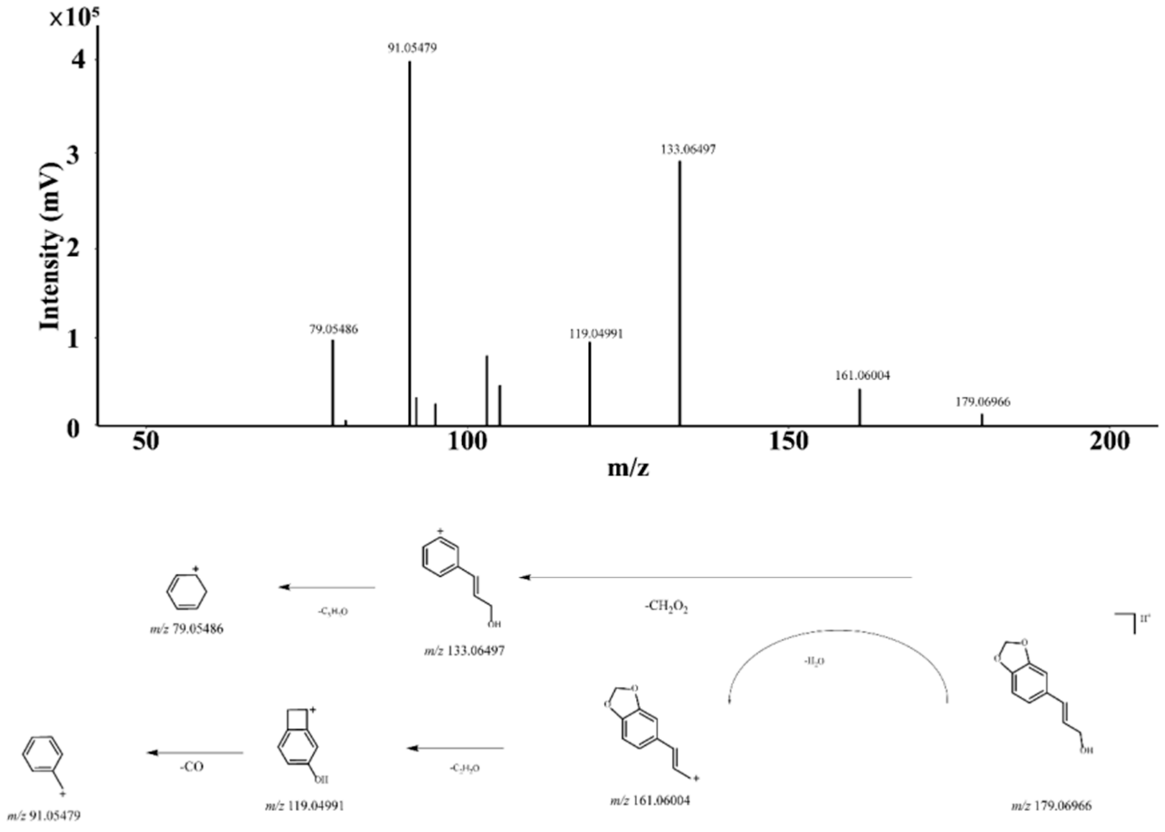

Figure.S6 The targeted MS/MS and fragmentation pattern of 3,4-Methylenedioxy cinnamyl alcohol

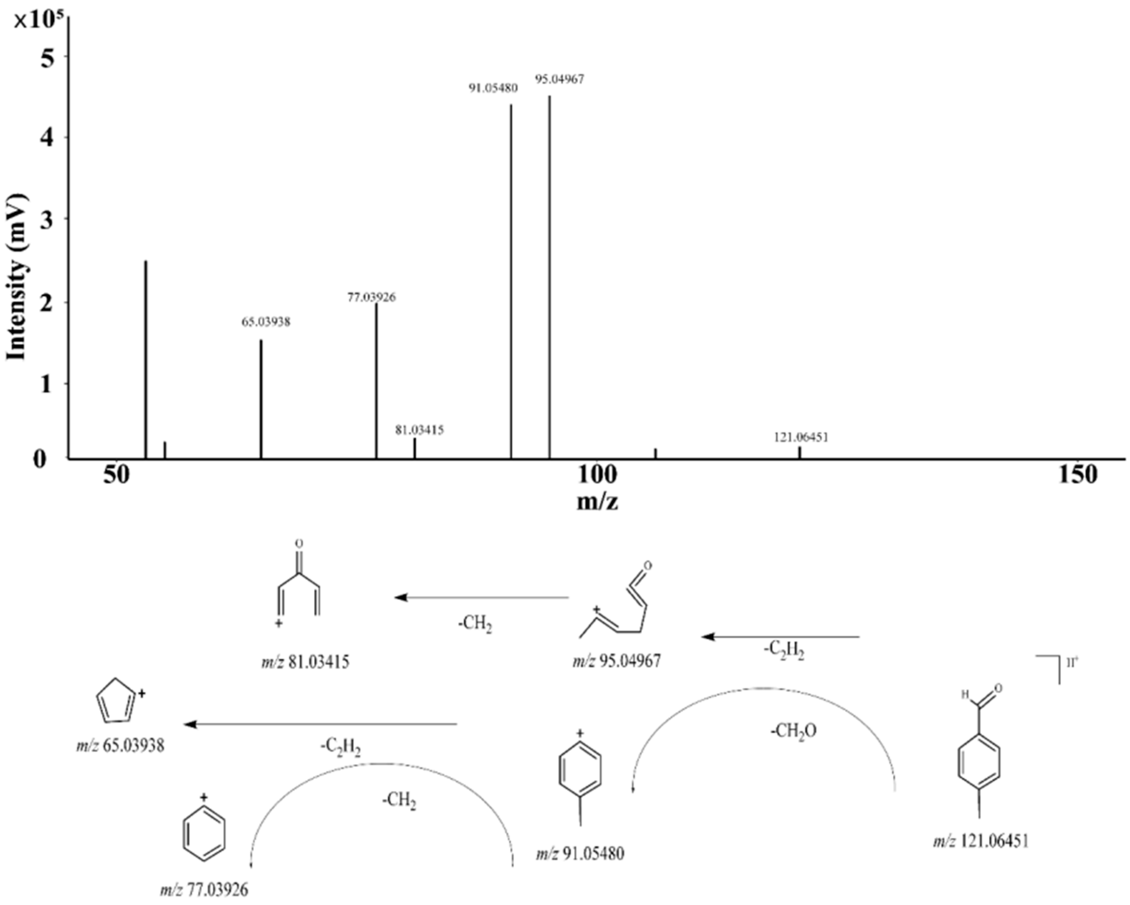

Figure.S7 The targeted MS/MS and fragmentation pattern of 4-Methylbenzaldehyde

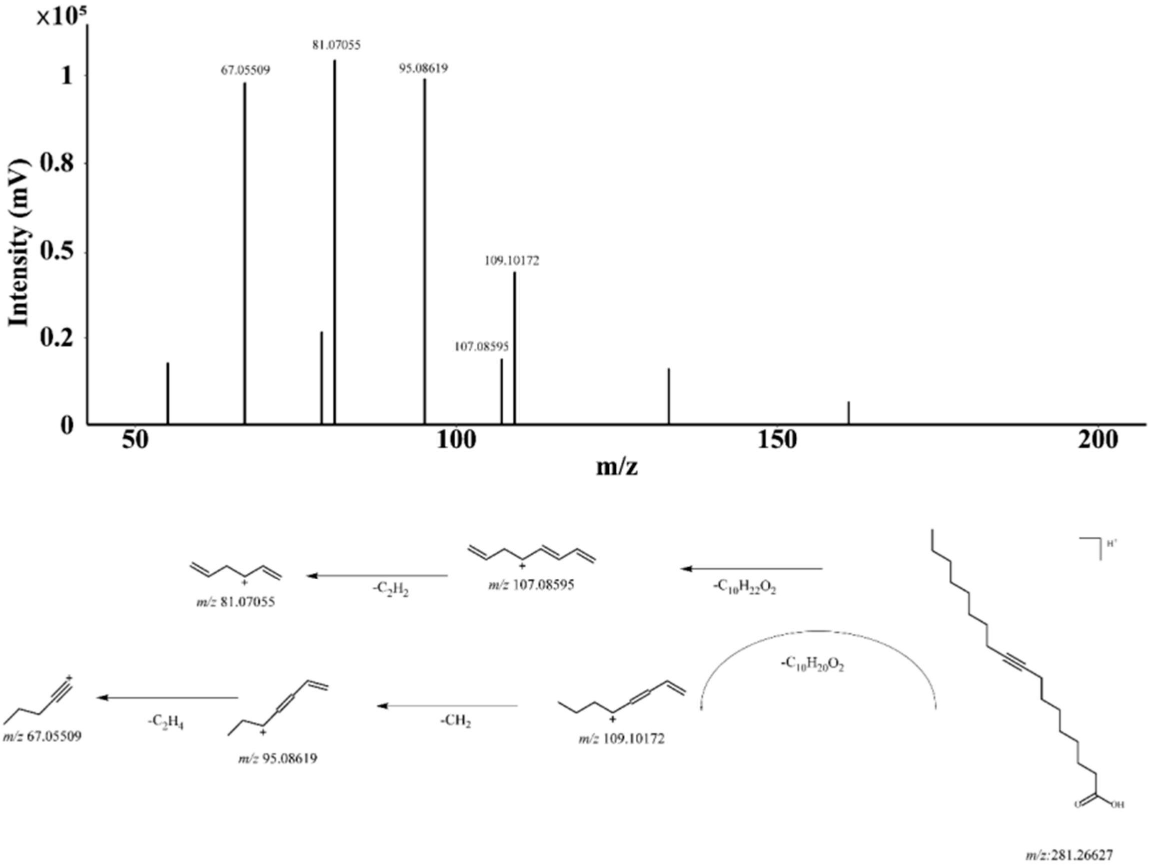

Figure.S8 The targeted MS/MS and fragmentation pattern of 9-Octadecynoic acid

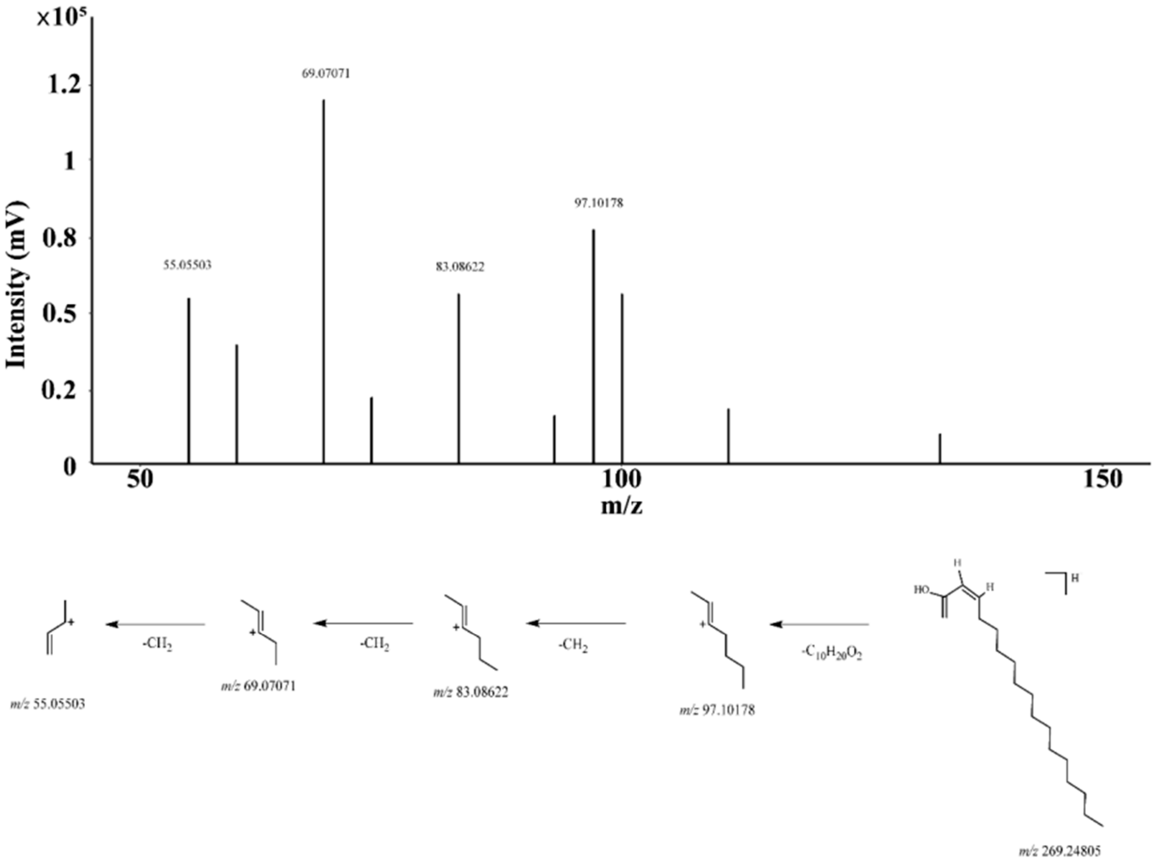

Figure.S9 The targeted MS/MS and fragmentation pattern of 10-Heptadecenoic Acid

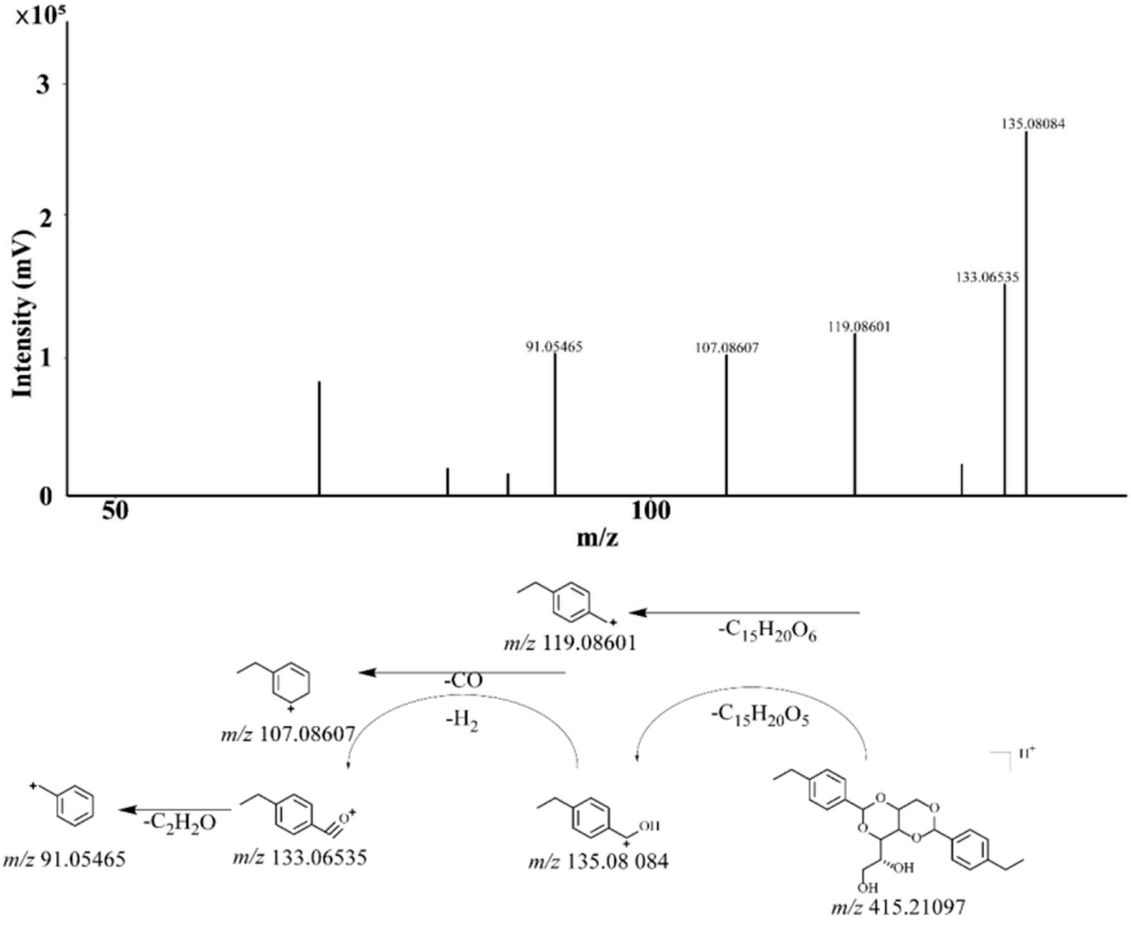

Figure.S10 The targeted MS/MS and fragmentation pattern of Bis(4-ethylbenzylidene)sorbitol

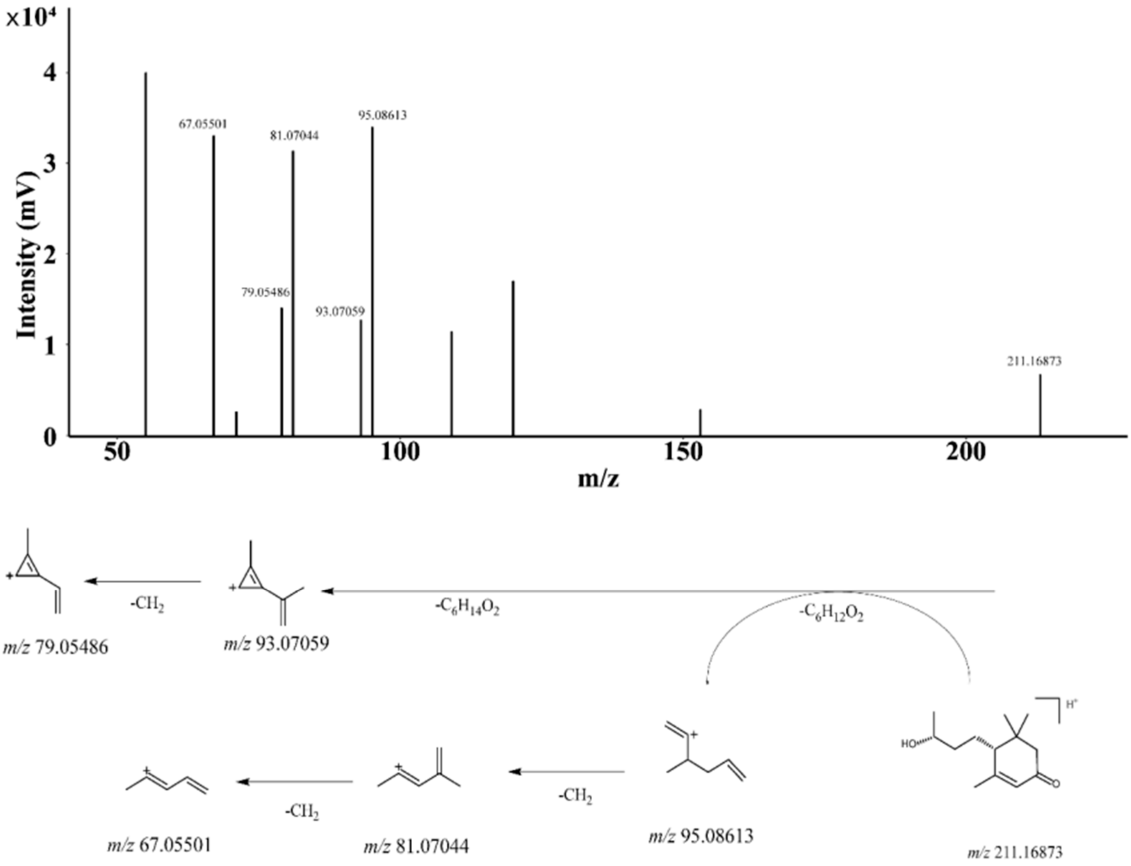

Figure.S11 The targeted MS/MS and fragmentation pattern of Blumenol C

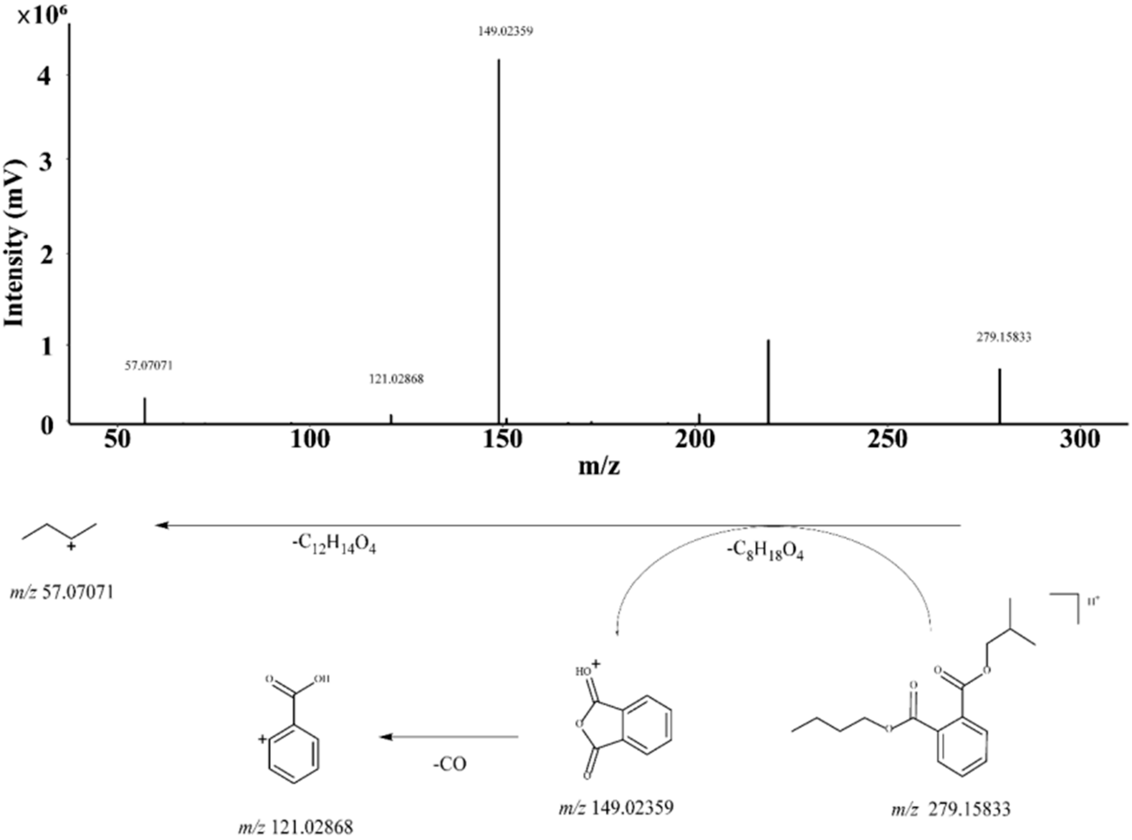

Figure.S12 The targeted MS/MS and fragmentation pattern of Butyl isobutyl phthalate

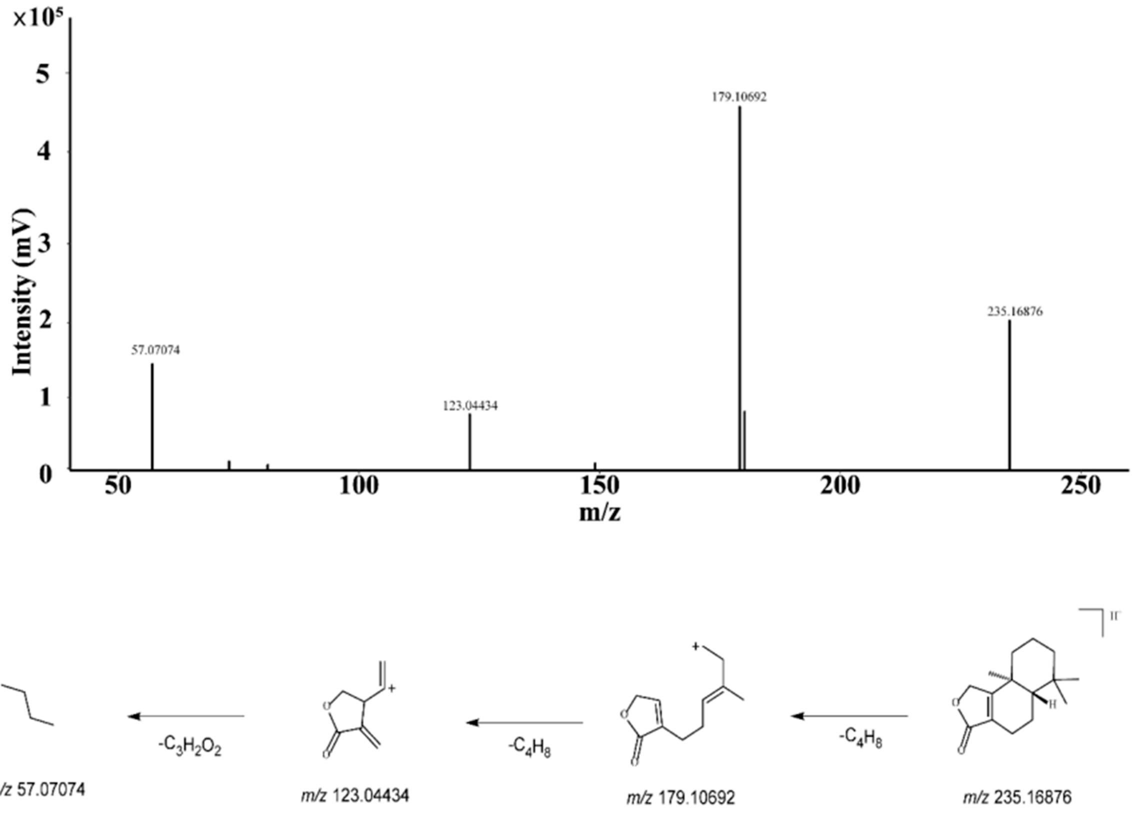

Figure.S13 The targeted MS/MS and fragmentation pattern of Confertifoline

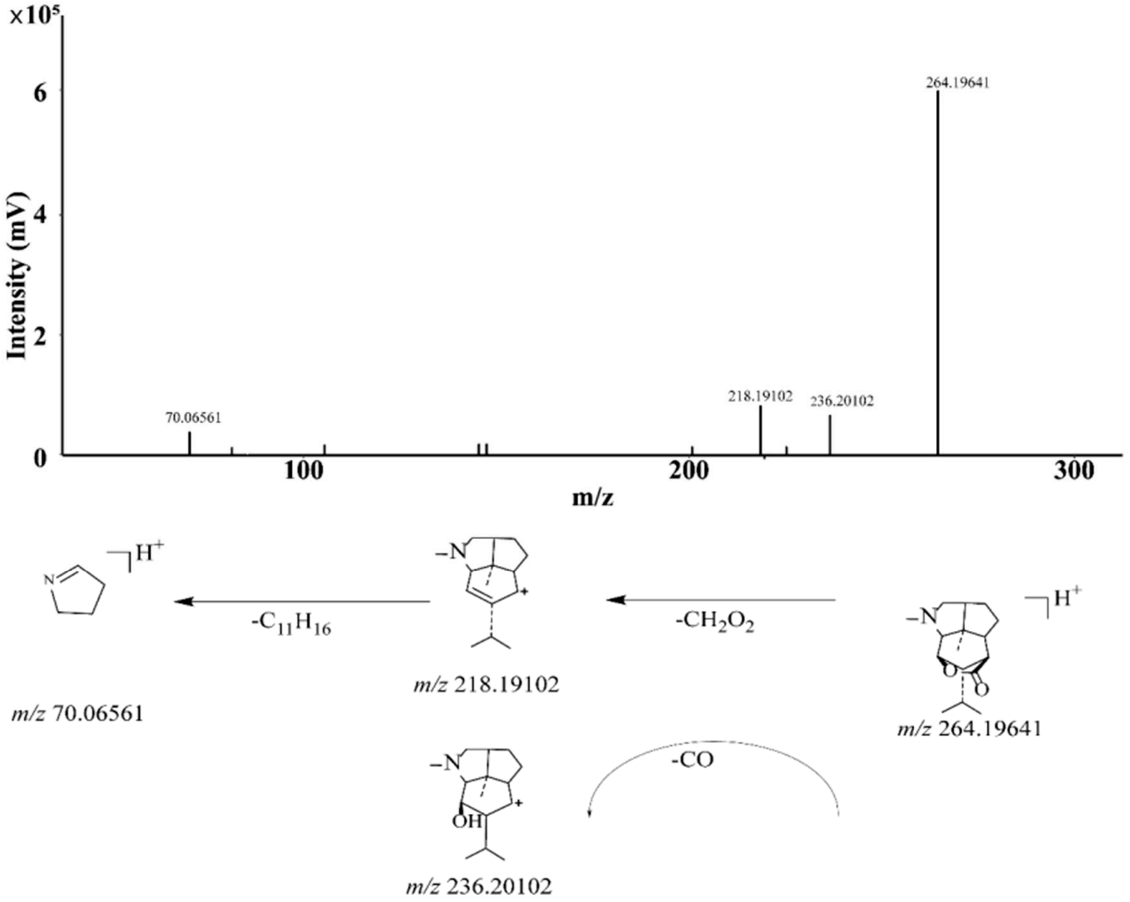

Figure.S14 The targeted MS/MS and fragmentation pattern of Dendrobine

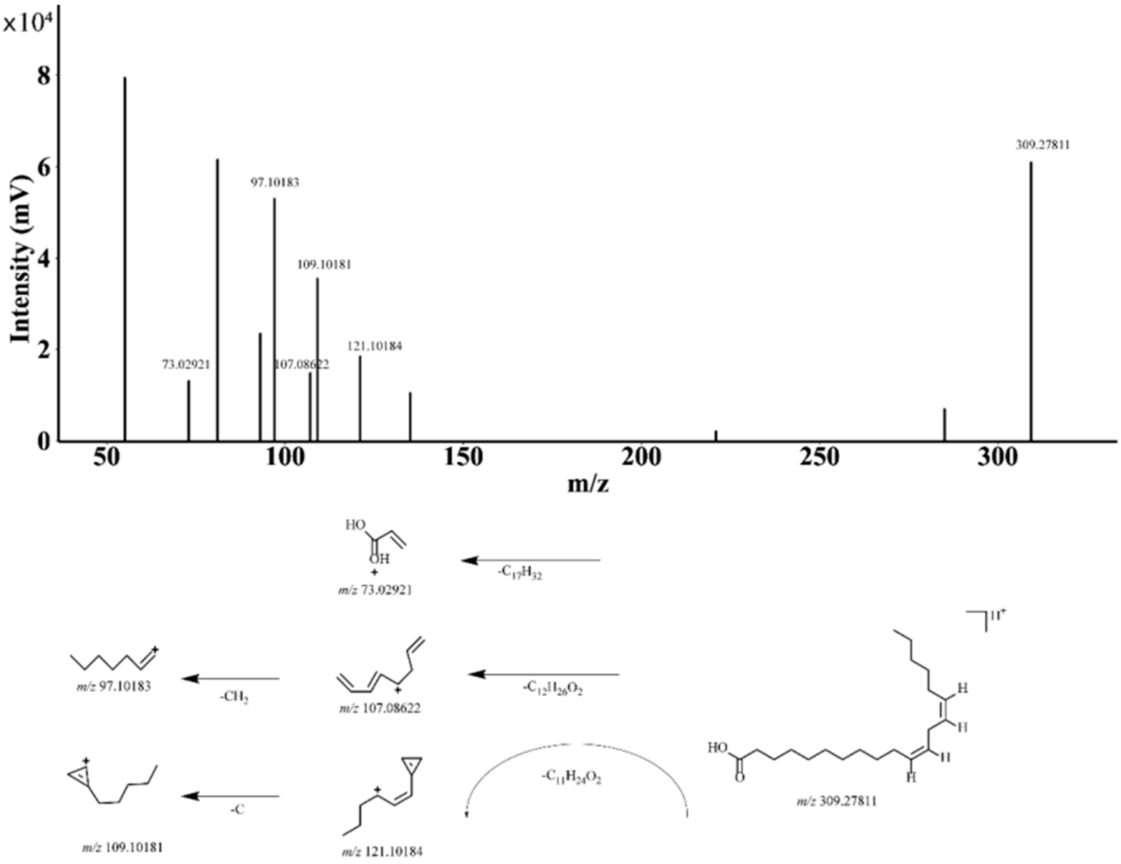

Figure.S15 The targeted MS/MS and fragmentation pattern of Eicosadienoic acid

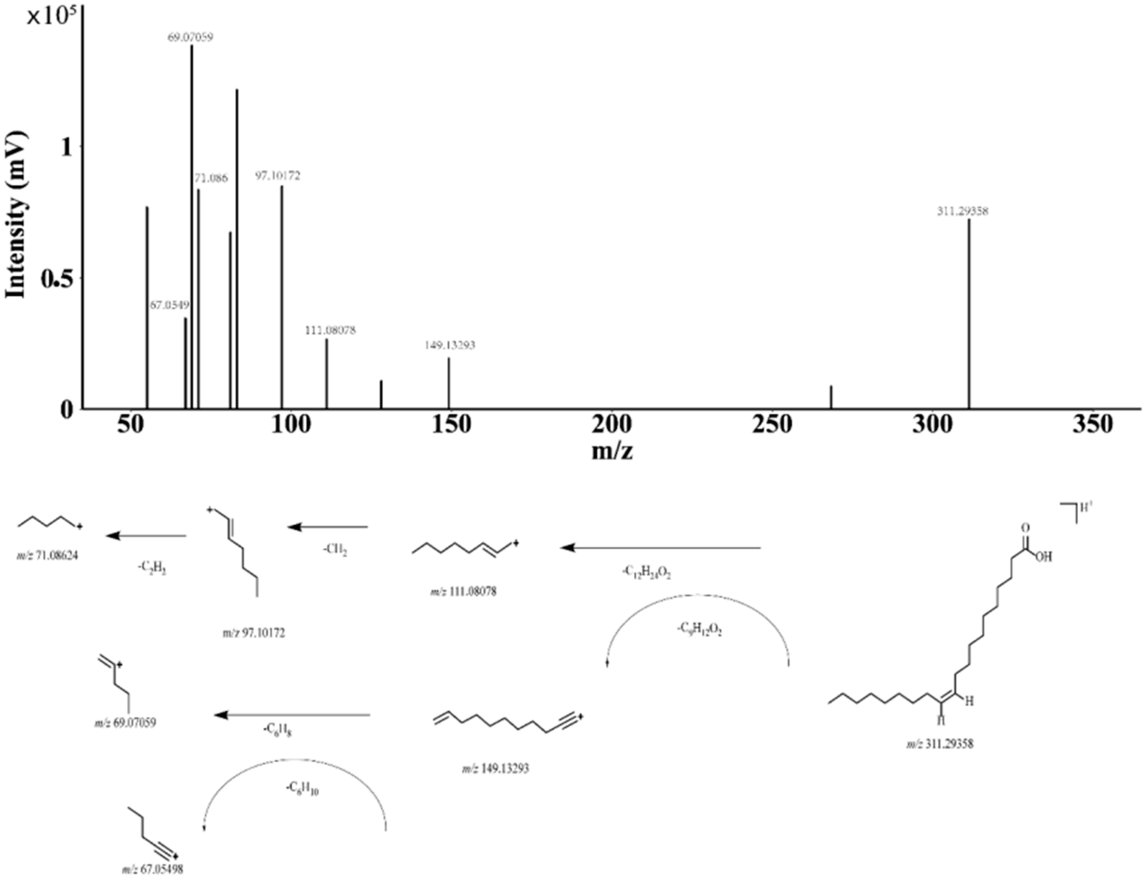

Figure.S16 The targeted MS/MS and fragmentation pattern of Eicosenoic acid

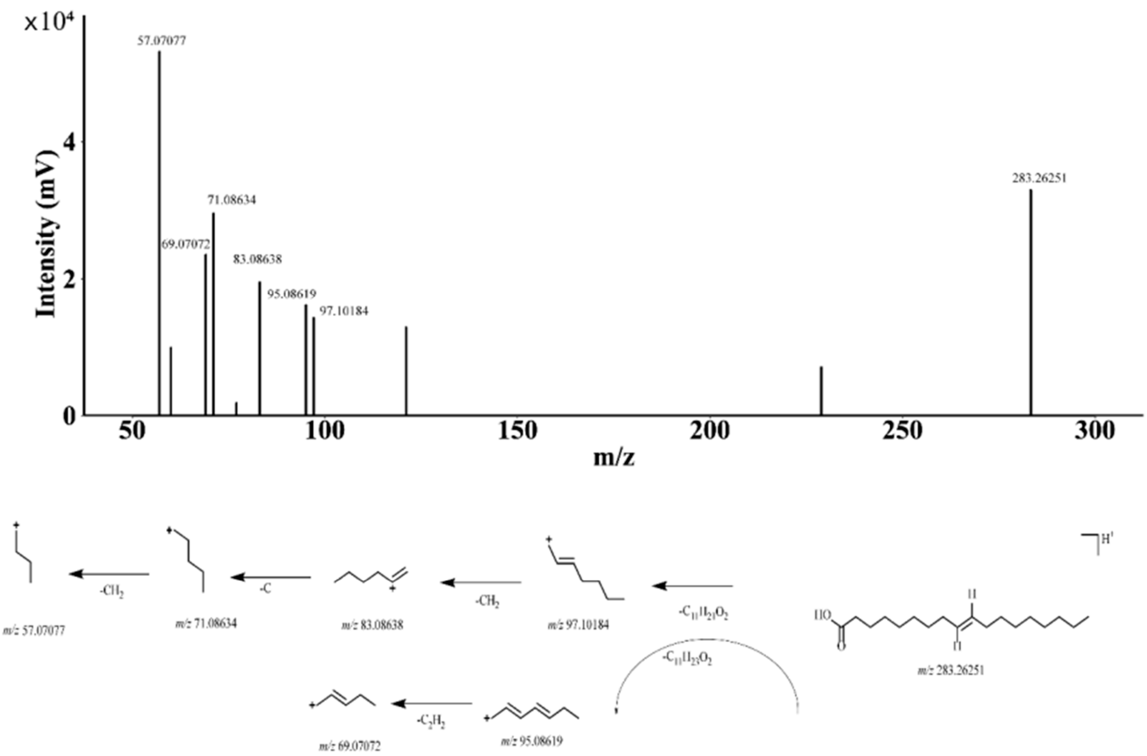

Figure.S17 The targeted MS/MS and fragmentation pattern of Elaidic Acid

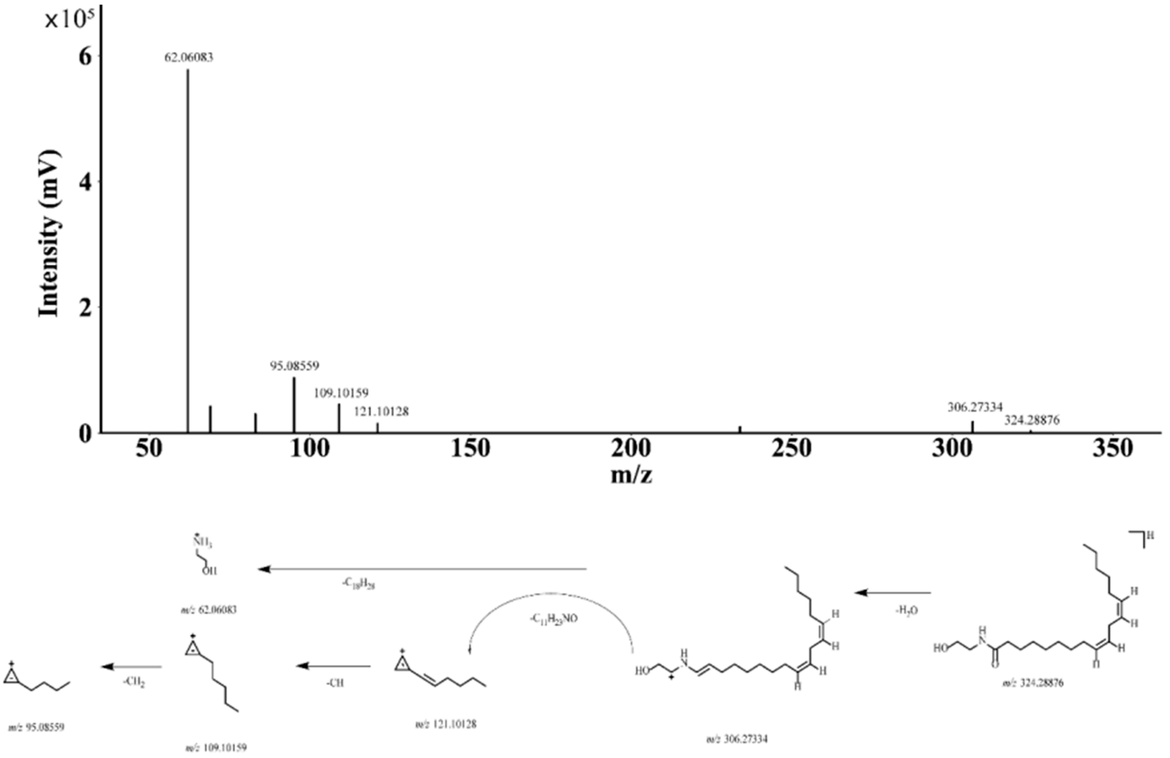

Figure.S18 The targeted MS/MS and fragmentation pattern of Linoleoyl ethanolamide

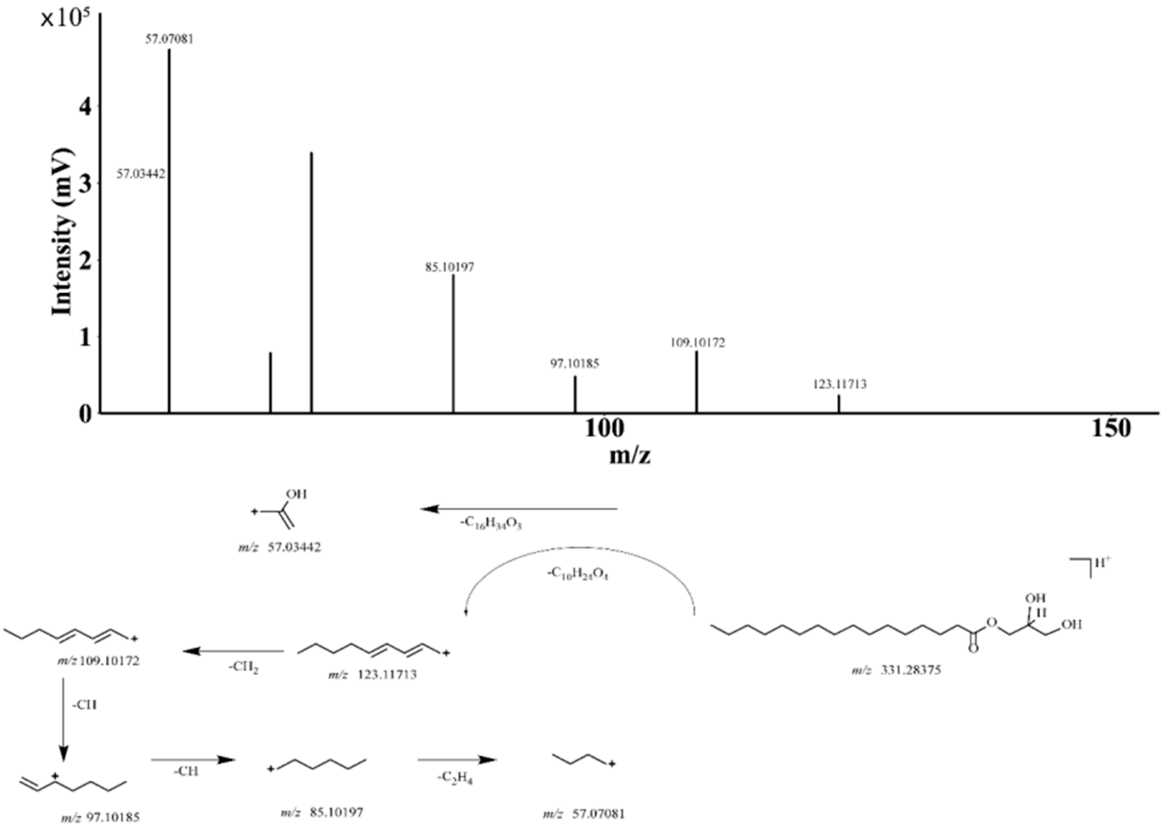

Figure.S19 The targeted MS/MS and fragmentation pattern of Monopalmitin

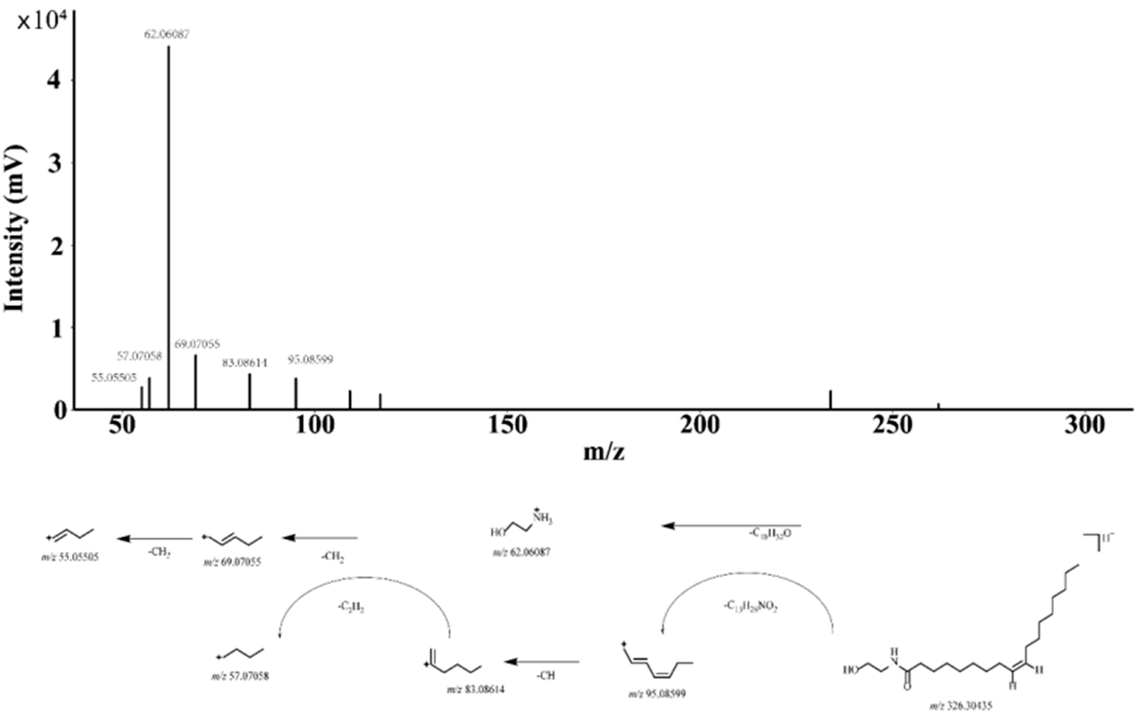

Figure.S20 The targeted MS/MS and fragmentation pattern of N-Oleoylethanolamine

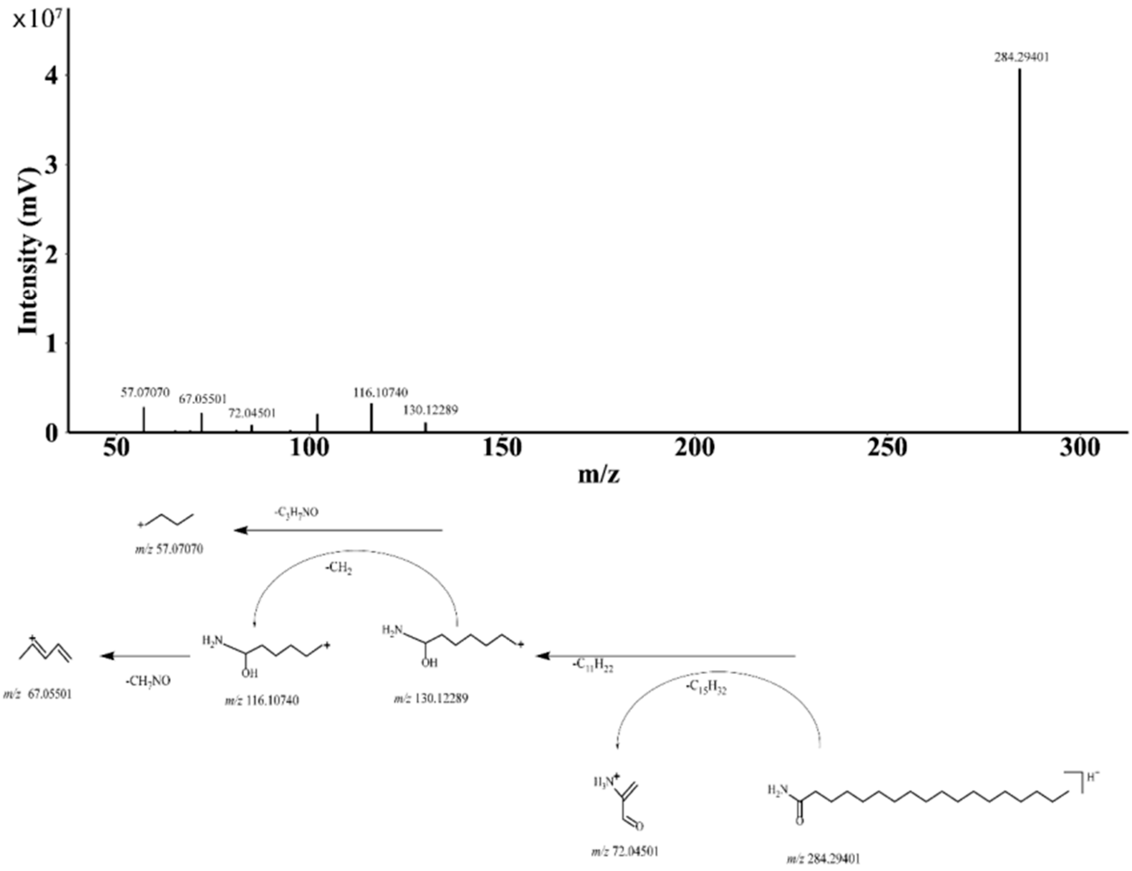

Figure.S21 The targeted MS/MS and fragmentation pattern of Octadecanamide

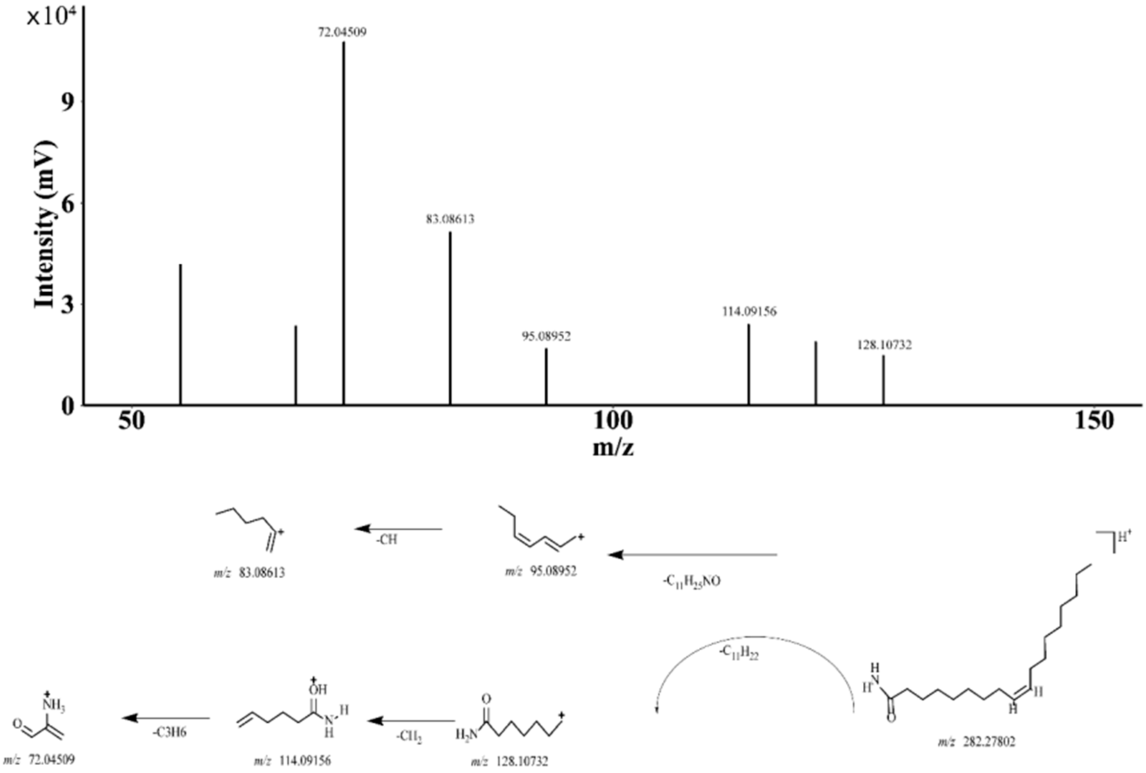

Figure.S22 The targeted MS/MS and fragmentation pattern of Oleamide

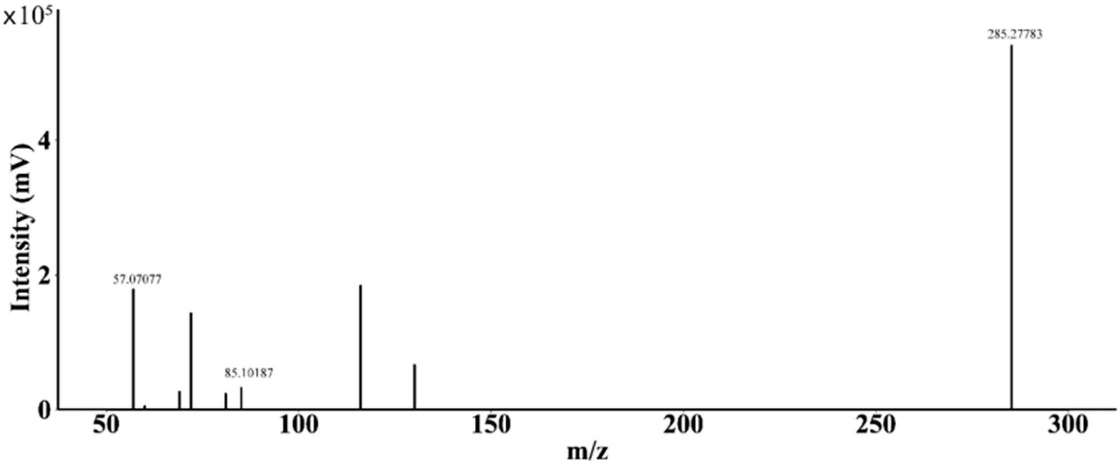

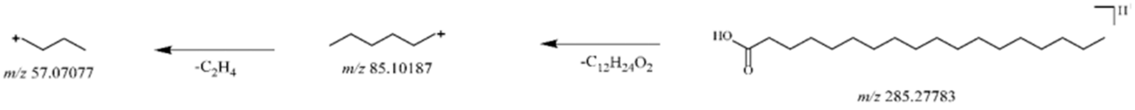

Figure.S23 The targeted MS/MS and fragmentation pattern of Stearic Acid

4. *Main Software and Databases*

Detailed information regarding the main software and databases used in the experiment can be found in Table S3.

Table S3 The information of the main software and database

**Software and database**	Version/URL
Analyst MD	Version 1.6.3
ChemDeaw	Version 20.0
Compound Discover	Version 3.2
Cytoscape	Version 3.10.0
Pubmed	https://pubmed.ncbi.nlm.nih.gov/
R (version: 4.1.1)	https://www.r-project.org/
Swiss Target Prediction	http://www.swisstargetprediction.ch/
Scifinders	https://scifinder.cas.org
WebGestalt	http://www.webgestalt.org/
Similarity Evaluation System of Chromatographic Fingerprint of Traditional Chinese Medicine	Version 2012
